# Lagrangian Submanifolds of Symplectic Structures Induced by Divergence Functions

**DOI:** 10.3390/e22090983

**Published:** 2020-09-03

**Authors:** Marco Favretti

**Affiliations:** Dipartimento di Matematica Tullio Levi-Civita, Università degli Studi di Padova, 35121 Padova, Italy; favretti@math.unipd.it

**Keywords:** canonical divergence, Lagrangian submanifolds, Morse family, constrained optimization, geometric phase transitions

## Abstract

Divergence functions play a relevant role in Information Geometry as they allow for the introduction of a Riemannian metric and a dual connection structure on a finite dimensional manifold of probability distributions. They also allow to define, in a canonical way, a symplectic structure on the square of the above manifold of probability distributions, a property that has received less attention in the literature until recent contributions. In this paper, we hint at a possible application: we study Lagrangian submanifolds of this symplectic structure and show that they are useful for describing the manifold of solutions of the Maximum Entropy principle.

## 1. Introduction

Information Geometry [1,2] provides a sound and fruitful framework for interpreting statistics using classical differential geometry notions [3]. A principal object in Information Geometry is the notion of contrast or divergence function, which (informally speaking) measures the degree of separation between two probability distributions [4,5,6]. The main thrust of divergence functions is that they allow to define a Riemannian structure on a finite dimensional submanifold *M* of probability distributions endowed with a dual coordinate system, with far reaching implications. A less-studied spin off of contrast function is the possibility of introducing a symplectic structure on the *square* of *M* by the pull-back of the canonical symplectic structure defined on the cotangent bundle $T^{*}M$. This procedure was introduced in 1995 in the pioneering paper [7], suggesting that symplectic geometry may have a natural role to play in statistics. In recent times there has been a renewed interest in possible applications of the symplectic structures introduced, as in [7] for example, to studying the analogies with the discrete Lagrangian mechanics (see in [8]) or the relations with completely integrable systems of Hamiltonian mechanics (see in [9,10]).

In this paper, we try to look at a possible role for Lagrangian submanifolds of the above-discussed symplectic structure on $M^{2}$ in the case that *M* is an *exponential family*M(h,k). Exponential families are prototypical examples of finite dimensional manifolds admitting a dually flat canonical structure defined by the *canonical divergence*, and they play a relevant role in information geometry and statistics [1,2]. For our argument, their importance is due to the fact that they represent the manifold of solutions of the variational problem associated to the Maximum Entropy Principle (MEP) with linear constraints ([11,12]). In some applications to statistical mechanics, e.g., in the descriptions of phase transitions in Ising spin systems, MEP with *nonlinear* constraints is considered, see, e.g., in [13,14,15]. In this case, the set of possible solutions has a richer structure, which is well captured by a Lagrangian submanifold of $T^{*}M\left(h,k\right)$. In this work, we are concerned with the Lagrangian submanifolds defined in the square of M(h,k) via the canonical pull-back hinted at above.

The structure of the paper is as follows. In Section 2, we recall the needed tools of Symplectic Geometry, and in Section 2.1 we review the canonical pull-back construction via divergence function construction exposed in [7]. In Section 3, we consider the special case of exponential families associated with MEP with nonlinear constraints.

## 2. Synopsis of Symplectic Geometry

We briefly recall the basic facts of symplectic geometry that are necessary for introducing our argument referring to classical textbooks for the proof of the results. A symplectic manifold (M,ω) is a smooth even-dimensional manifold *M* equipped with a non-degenerate, closed two-form ω (dω=0, where *d* is the external derivation operator). A submanifold *L* of *M* is a *Lagrangian* submanifold if 2dimL=dimM and the two-form restricted to *L* is vanishing, $\omega_{|L}=0$. A prototypical example of symplectic manifold is the cotangent bundle $T^{*}S$ of a manifold *S*. If $x=(x^{1},\ldots ,x^{n})$ are local coordinates on *S*, and (x,λ) are local coordinates on $T^{*}S$, then the Liouville one-form $\theta_{c}$ on $T^{*}S$ has the local expression $\theta_{c}=\lambda_{i}dx^{i}$ (summation over repeated indices is understood) and the symplectic two form is

(1) $$\omega =d\theta_{c}=d\lambda_{i}\wedge dx^{i}.$$

A classical theorem of Darboux says that every symplectic manifold (M,ω) admits an atlas of local coordinates (x,λ) such that locally ω has the representation (1). A relevant example of Lagrangian submanifold of $T^{*}S$ is the graph of the differential of a function g:S→R, that is,

$$L_{g}=\left\{\left(x,\lambda \left(x\right)\right)\in T^{*}S\;:\;\lambda \left(x\right)=dg\left(x\right),\;x\in S\right\}.$$

Note that $L_{g}$ is a *n*-dimensional submanifold which is *transversal* to the fibers of the fibration $\pi :T^{*}S\to \;S$, that is, its tangent bundle $TL_{g}$ is transversal to the vertical bundle kerTπ.

According to a theorem of Maslov–Hormander ([16,17]), a general (i.e. not necessarily trasversal) Lagrangian submanifold of $T^{*}S$ can be locally described as the graph of a smooth function *G* depending on extra parameters. Let us sketch briefly this construction along the lines of the works in [18,19].

Let *U* be a *k*-dimensional manifold called supplementary manifold, and let G:S×U→R be a smooth function whose representation in a local chart is G(x,u). We define the *critical set* of *G* as (we use the notation ${\left(G_{x}\right)}_{i}=\partial G/\partial x^{i}$ and ${\left(G_{xy}\right)}_{ij}=\partial^{2}G/\partial x^{i}\partial y^{j}$) for partial derivatives)

(2) $$E=\{\left(x,u\right)\;:\;G_{u}\left(x,u\right)=0\}.$$

If $dG_{u}$ has maximal rank over E, that is,

(3) $$\mathrm{rk}\;dG_{u}=\mathrm{rk}(G_{xu}\;G_{uu})=k\;\mathrm{for}\;\mathrm{all}\;\left(x,u\right)\in E$$

then *G* is called *Morse family* and the following $\Lambda_{G}$ is a Lagrangian submanifold of $T^{*}S$,

(4) $$\Lambda_{G}=\left\{\left(x,G_{x}\left(x,u\right)\right)\in T^{*}S\;\mathrm{where}\;\left(x,u\right)\in E\right\}.$$

If there are no extra parameters k=0, then $\Lambda_{G}$ is the graph of a differential and thus $\Lambda_{G}$ is a transversal submanifold. Note that the above rank condition (3) can be satisfied if the square submatrix $G_{uu}$ has maximal rank, i.e., $\det G_{uu}\neq 0$ on E. In this case, by the implicit function theorem there exist a locally defined function u=u(x) such that E is the graph of *u* and setting $\hat{G}\left(x\right)=G\left(x,u\left(x\right)\right)$ we have that

$${\hat{G}}_{x}\left(x\right)=G_{x}\left(x,u\left(x\right)\right)+G_{u}\left(x,u\left(x\right)\right)u_{x}\left(x\right)=G_{x}\left(x,u\left(x\right)\right)\;\mathrm{for}\;\mathrm{all}\;\left(x,u\right)\in E.$$

Therefore, where $\det G_{uu}\neq 0$ on E, all the parameters *u* can be eliminated and $\Lambda_{\hat{G}}$ is locally transversal to the fibers. The set of points of *S* where $\det G_{uu}\left(x,u\right)=0$ for (x,u)∈E is called the *caustic* of $\Lambda_{G}$. These are the points where the Lagrangian submanifold is *tangent* to the fibers of $\pi :T^{*}S\to S$ and trasversality is lost.

### 2.1. Symplectic Structures Defined by Divergence Functions

Given a smooth *n*-dimensional manifold, *M*, let us denote with $M^{2}=M\times M$ the square of *M* and with $\Delta_{M}\subset M^{2}$ the diagonal of $M^{2}$. We will use local coordinates $x=(x^{1},\ldots ,x^{n})$ on *M* and $\left(x,y\right)=\left(x^{1},\ldots ,x^{n},y^{1},\ldots ,y^{n}\right)$ on $M^{2}$.

Let $D:M^{2}\to \left[0,+\infty \right)$ be a smooth non-negative function whose representation in a local chart is D(x,y)≥0. We use the notations

$${\left(D_{x}\right)}_{i}=\frac{\partial D}{\partial x^{i}},\;{\left(D_{y}\right)}_{j}=\frac{\partial D}{\partial y^{j}},\;{\left(D_{yx}\right)}_{ji}=\frac{\partial }{\partial y^{j}}\left(\frac{\partial D}{\partial x^{i}}\right)=-\phi_{ji}$$

for first and second order derivatives of *D*. The function *D* is a *yoke* (see [7] ) if the following conditions hold and *D* is a *divergence* (see [8]) if (iii) below holds on the whole $M^{2}$.

(i) D=0 only on $\Delta_{M}$
(ii) $D_{x}=0$ and $D_{y}=0$ on $\Delta_{M}$
(iii) $\phi =-D_{xy}$ is positive definite on $\Delta_{M}$

thus points of $\Delta_{M}$ are minima of *D*. A divergence function act as a pseudo-distance but it does not satisfy the symmetry nor the triangle inequality conditions. In [7], the following fibered map $F_{D}:M^{2}\to T^{*}M$ over *M* is considered, whose representation in a local chart is

(5) $$F_{D}\left(x,y\right)=\left(x,D_{x}\left(x,y\right)\right).$$

By condition (iii) above there exist a neighborhood *W* of $\Delta_{M}$, where $F_{D}$ has a smooth inverse

$$F_{D}^{-1}\left(x,\lambda \right)=\left(x,y\left(x,\lambda \right)\right).$$

Using the local diffeomorphism $F_{D}$ a symplectic structure $\left(W,\omega_{D}\right)$ is defined in [7] via the pull-back $\omega_{D}=F_{D}^{*}\omega$ of the canonical two form (1) on $T^{*}M$. The local form of $\omega_{D}$ can be computed as follows,

(6) $$\omega_{D}=F_{D}^{*}\omega =F_{D}^{*}\left(d\theta_{c}\right)=d\left(F_{D}^{*}\theta_{c}\right)=d\left({\left(D_{x}\right)}_{i}dx^{i})\right)$$

thus (see Section 3.2 in [7])

$$\omega_{D}=\frac{\partial^{2}D}{\partial x^{j}\partial x^{i}}dx^{j}\wedge dx^{i}+\frac{\partial^{2}D}{\partial y^{j}\partial x^{i}}dy^{j}\wedge dx^{i}=-\phi_{ji}dy^{j}\wedge dx^{i}$$

because the first term $\partial^{2}D/\partial x^{j}\partial x^{i}$ is symmetric in the i,j indices. For the applications that we have in mind of the above theory, we will assume in (iii) above that $-D_{yx}$ is positive definite on the *whole* $M^{2}$ so that $F_{D}$ is a global diffeomorphism.

Simple examples of Lagrangian submanifolds of $M^{2}$ with respect to $\omega_{D}$ are (with a little abuse of notation) the *n*-dimensional submanifolds $M_{x}=M\times \left\{y\right\}\approx M$, which are also transversal to the fibers of $\pi_{1}:M^{2}\to M$, $\pi_{1}\left(x,y\right)=x$. Moreover, as $\omega_{D}\left(u,u\right)=0$, $\Delta_{M}$ is also a Lagrangian submanifold.

Note also that (6) implies that $F_{D}$ is a symplectomorphism, thus $L=F_{D}^{-1}\left(\Lambda \right)$ is a Lagrangian submanifold of $M^{2}$ whenever $\Lambda \subset T^{*}M$ is a Lagrangian submanifold. In this paper, we will be mainly concerned with the study of Lagrangian submanifolds of $M^{2}$ defined in this way.

In the following Section 2.2, we will compute the above introduced objects for the relevant case of exponential families of probability distributions and canonical divergence.

In [7], the Hamiltonian $H:T^{*}M\to \left[0,+\infty \right)$ associated to a divergence function is defined as $H=D\circ F_{D}^{-1}$ and locally it has the form

(7) H(x,λ)=D(x,y(x,λ)).

### 2.2. Canonical Divergence and Exponential Families

In this section, we recall the basic definitions of exponential family and canonical divergence, as described, e.g., in [1,2]. Let (X,B,dx) be a probability space, where *X* may be a discrete set or $X=R^{k}$. We stipulate that in case of a discrete set the integrals over *X* with respect to the measure dx are substituted by summations. Let

$$P\left(X\right)=\{p:X\to \left[0,+\infty \right),\;p\left(x\right)\geq 0,\int_{X}pdx=1\}$$

and suppose that q∈P(X) for suitable *k*, where $q\left(x\right)=e^{k(x)}>0$. Consider *n* independent *observables*

$$h:X\to R^{n},\;\mathrm{rk}\;dh\left(x\right)=n\;\forall \;x\in X$$

and define the related *free energy* $\psi :\Theta \subset R^{n}\to R$ as (here $\theta \cdot h=\theta^{i}h_{i}$)

(8) $$e^{\psi (\theta )}=\int_{X}e^{\theta \cdot h(x)+k(x)}dx.$$

The *n* real numbers $\theta^{i}$ are called *canonical* parameters. They define uniquely a probability distribution p(·;θ) which belongs to the *exponential family* defined by h,k,

(9) $$M\left(h,k\right)=\{\;p\left(x;\theta \right)=e^{\theta \cdot h(x)+k(x)-\psi (\theta )},\;\theta \in \Theta \}\subset P\left(X\right).$$

The relevant fact is that M(h,k) is a *n*-dimensional submanifold of the infinite dimensional set P(X) and that the canonical parameters θ are local coordinates. Note that q∈M(h,k) as ψ(0)=0 and q(x)=p(x;0). Another system of local coordinates is provided by the so-called *expectation* parameters defined by

$$\eta =\psi_{\theta }\left(\theta \right)=E_{p_{\theta }}\left[h\right]=\int_{X}h\left(x\right)p\left(x;\theta \right)dx.$$

As ψ is a convex function, the gradient map $\psi_{\theta }\left(\theta \right)=\eta$ is globally invertible with inverse $\theta =\hat{\theta }\left(\eta \right)$, which is also a gradient map $\hat{\theta }\left(\eta \right)=\varphi_{\eta }\left(\eta \right)$, where

(10) $$\varphi \left(\eta \right)=\hat{\theta }\left(\eta \right)\cdot \eta -\psi \left(\hat{\theta }\left(\eta \right)\right)$$

is the Legendre transform of ψ (see, e.g., in [1]). We will denote with p(x;η) the point in M(h,k) associated to η. The Kullback–Leibler divergence is defined for general $\left(p,\tilde{p}\right)$ in $P{\left(X\right)}^{2}$ as

$$D_{KL}\left(p,\tilde{p}\right)=\int_{X}p\left(x\right)\log\frac{p(x)}{\tilde{p}\left(x\right)}dx.$$

The restriction of $D_{KL}$ to $M{\left(h,k\right)}^{2}$, the square of M(h,k), $D_{KL}:M{\left(h,k\right)}^{2}\to \left[0,+\infty \right)$ is called *canonical* divergence. It can be shown (see in [1]) that when M(h,k) is *referred to the coordinates* (η,θ), $D_{KL}$ has the local representation

(11) D(η,θ)=φ(η)+ψ(θ)−η·θ.

Note that as p(·;θ)=q for θ=0

(12) $$D_{KL}\left(p,q\right)=D_{KL}\left(p\left(\cdot ;\eta \right),p\left(\cdot ;0\right)\right)=\varphi \left(\eta \right)+\psi \left(0\right)-\eta \cdot 0=\varphi \left(\eta \right).$$

A key object is the map $F_{D}$ introduced in (5) associated to M(h,k) and the canonical divergence (11). It has the local form in coordinates (η,θ), see (5) and (11),

(13) $$F_{D}\left(\eta ,\theta \right)=\left(\eta ,D_{\eta }\right)=\left(\eta ,\varphi_{\eta }\left(\eta \right)-\theta \right),$$

with the *explicit* inverse, using local coordinates (η,λ) in $T^{*}M\left(h,k\right)$,

(14) $$F_{D}^{-1}\left(\eta ,\lambda \right)=\left(\eta ,\theta \left(\eta ,\lambda \right)\right)=\left(\eta ,\varphi_{\eta }\left(\eta \right)-\lambda \right)=\left(\eta ,\hat{\theta }\left(\eta \right)-\lambda \right).$$

A simple but elegant result of the above-introduced framework is the following.

**Proposition** **1.** *Let $\Lambda_{G}$ be a Lagrangian submanifold of $T^{*}M\left(h,k\right)$ described by the Morse family G(η,u) as in (4). Then, $L_{S}=F_{D}^{-1}\left(\Lambda_{G}\right)$ is a Lagrangian submanifold of $M{\left(h,k\right)}^{2}$ described by the Morse family S(η,u)=φ(η)−G(η,u).*

**Proof.**  From (4) we have that $\lambda =G_{\eta }\left(\eta ,u\right)$ on $\Lambda_{G}$ and from (14)

$$F_{D}^{-1}\left(\Lambda_{G}\right)=\left\{\left(\eta ,\theta \right)=\left(\eta ,\varphi_{\eta }\left(\eta \right)-G_{\eta }\left(\eta ,u\right)\right)=\left(\eta ,S_{\eta }\left(\eta ,u\right)\right),\;\left(\eta ,u\right)\in E\right\}$$

where S(η,u)=φ(η)−G(η,u). Moreover, as $S_{u}\left(\eta ,u\right)=G_{u}\left(\eta ,u\right)$ the critical set E in (2) is the same. □

As a consequence of the above proposition, if $\Lambda_{G}$ is transversal to the fibers of $T^{*}M\left(h,k\right)$ (no extra parameters *u*), then its image in $M{\left(h,k\right)}^{2}$ is transversal to the fibers of $\pi_{1}$.

Another interesting consequence is that the zero section of the cotangent bundle $T^{*}M\left(h,k\right)$, locally represented as Z={(η,0):η∈E}, is mapped by $F_{D}^{-1}$ into

$$Z_{0}=F_{D}^{-1}\left(Z\right)=\left\{\left(\eta ,\hat{\theta }\left(\eta \right)\right)\;:\;\eta \in E\right\}$$

which is contained into $D^{-1}\left(0\right)$, the zero-level set of the canonical divergence. Indeed, from (10) and (11) we have that

(15) $$D\left(\eta ,\hat{\theta }\left(\eta \right)\right)=\varphi \left(\eta \right)+\psi \left(\hat{\theta }\left(\eta \right)\right)-\eta \cdot \hat{\theta }\left(\eta \right)=\varphi \left(\eta \right)-\varphi \left(\eta \right)\equiv 0$$

thus $Z_{0}\subset D^{-1}\left(0\right)$ in the general case and $Z_{0}=D^{-1}\left(0\right)$ if n=1. For later use, we compute from (7) the Hamiltonian associated to the canonical divergence

$$H\left(\eta ,\lambda \right)=D\circ F_{D}^{-1}\left(\eta ,\lambda \right)=\varphi \left(\eta \right)+\psi \left(\hat{\theta }\left(\eta \right)-\lambda \right)-\eta \cdot \left(\hat{\theta }\left(\eta \right)-\lambda \right).$$

We set for the sake of simplicity $\hat{\theta }\left(\eta \right)=\hat{\theta }$ and we compute from (8) the free energy $\psi (\hat{\theta }\left(\eta \right)-\lambda )$

(16) $$\begin{array}{ccc} e^{\psi (\hat{\theta }-\lambda )} & = & \int_{X}e^{(\hat{\theta }-\lambda )\cdot h+k}dx=\int_{X}e^{\left(\hat{\theta }-\lambda \right)\cdot h+k\;+\psi \left(\hat{\theta }\right)-\psi \left(\hat{\theta }\right)}dx\\ & = & e^{\psi (\hat{\theta })}\int_{X}e^{-\lambda \cdot h}e^{\hat{\theta }\cdot h+k-\psi \left(\hat{\theta }\right)}dx=e^{\psi (\hat{\theta })}E_{p_{\hat{\theta }}}\left[e^{-\lambda \cdot h}\right]. \end{array}$$

Using (15) and (16), the Hamiltonian can be written using relation (10) as

(17) $$\begin{array}{ccc} H(\eta ,\lambda ) & = & \varphi \left(\eta \right)+\psi \left(\hat{\theta }\right)+\ln E_{p_{\hat{\theta }}}\left[e^{-\lambda \cdot h}\right]-\eta \cdot \hat{\theta }+\eta \cdot \lambda \\ & = & \ln E_{p_{\hat{\theta }}}\left[e^{-\lambda \cdot h}\right]+\eta \cdot \lambda . \end{array}$$

It is interesting to investigate more in detail the structure of the Lagrangian submanifold $L_{S}=\;F_{D}^{-1}\left(\Lambda_{G}\right)\subset M{\left(h,k\right)}^{2}$ by studying the form of the two probability distributions $F_{D}^{-1}\left(\eta ,\lambda \right)=\;\left(\eta ,\hat{\theta }-\lambda \right)$ in $L_{S}$ associated to the coordinates respectively η and $\hat{\theta }-\lambda$. We compute from (9)

$$p\left(x;\eta \right)=e^{\hat{\theta }\cdot h\left(x\right)+k\left(x\right)-\psi \left(\hat{\theta }\right)}$$

and using (17)

(18) $$\begin{array}{ccc} p(x;\hat{\theta }-\lambda ) & = & e^{\left(\hat{\theta }-\lambda \right)\cdot h+k-\psi \left(\hat{\theta }-\lambda \right)}\\ & = & e^{\hat{\theta }\cdot h-\lambda \cdot h+k-\psi \left(\hat{\theta }\right)-\ln E_{p_{\hat{\theta }}}\left[e^{-\lambda \cdot h}\right]}\\ & = & p\left(x;\eta \right)\frac{e^{-\lambda \cdot h(x)}}{E_{p_{\hat{\theta }}}\left[e^{-\lambda \cdot h}\right]}\;. \end{array}$$

Note that setting

$$p\left(x;\lambda \right)=\frac{e^{-\lambda \cdot h(x)}}{Z(\lambda )}=\frac{e^{-\lambda \cdot h(x)}}{\int_{X}e^{\lambda \cdot h(x)dx}}$$

relation (18) can be given the form

(19) $$p\left(x;\hat{\theta }-\lambda \right)=\frac{p\left(x;\eta \right)e^{-\lambda \cdot h(x)}}{\int_{X}p\left(x;\eta \right)e^{-\lambda \cdot h(x)}dx}=\frac{p(x;\eta )p(x;\lambda )}{\int_{X}p\left(x;\eta \right)p\left(x;\lambda \right)dx}\;.$$

We will give an interpretation of this relation in the case of discrete probability distributions in Section 3.2 below.

## 3. Application to Maximum Entropy Principle with Nonlinear Constraints and Phase Transitions

A relevant application of the above-introduced framework concerns the use of the Maximum Entropy Principle with *nonlinear* constraints. Let us consider a physical system *X* whose description is given in terms of a probability distribution q∈P(X). The Maximum Entropy Principle (E.T. Jaynes, see in [11,12]) is a general inference procedure that allows to update an initial probability distribution *q* on the basis of subsequent information on the system represented by the average values $E_{p}\left[h\right]$ of some observables *h* of interest for the system. The sought distribution *p* is the one that minimizes the relative entropy $D_{KL}\left(p,q\right)$ on the set of the distributions which satisfy the constraints on $E_{p}\left[h\right]$. From a mathematical point of view, we are faced with a constrained extremization problem to be solved below using the Lagrange multipliers method.

We will see that the set of solutions for different values of the constraints defines a Lagrangian submanifold of a cotangent space of a manifold M(h,k). We are interested in describing the corresponding Lagrangian submanifold in $M{\left(h,k\right)}^{2}$.

This section has a pedagogical character, so for the sake of simplicity we will avoid technicalities and assume that X={1,…,n} is a discrete space and that there is only one observable of interest defined by assigning $h=(h_{1},\ldots ,h_{n})$. The case of *k* observables can be dealt with along the same lines with no extra effort. The case of a continuous space $X\subset R^{n}$ presents more technical difficulties and it is considered in [20].

Let $q_{i}=e^{k_{i}}\in P\left(X\right)$ be the a priori distribution describing *X*. The Kullback–Leibler divergence is called *relative entropy* in this setting and has the form

$$D\left(p,q\right)=\sum_{i}p_{i}\ln\frac{p_{i}}{q_{i}}.$$

**Figure 1:**
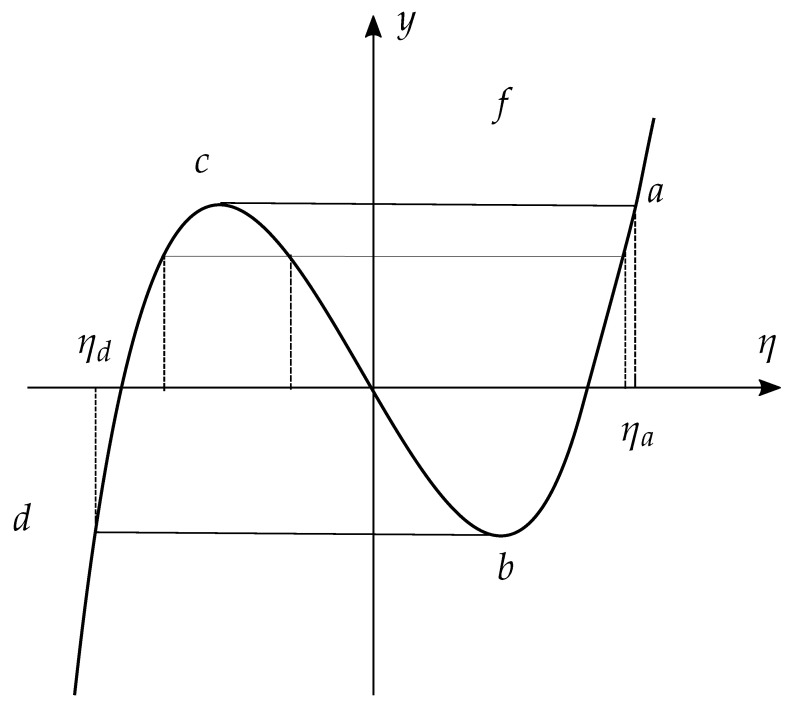
Plot of $y=f\left(\eta \right)=\eta \left(\eta^{2}-a^{2}\right)$. Points b,c correspond to points where $f^{\prime }\left(\eta \right)=0$.

Let f:R→R be a smooth globally non-invertible function (think for example of a cubic $f\left(x\right)=x\left(x^{2}-a^{2}\right)$ for a∈R, see Figure 1 below). We look for the minima of *D* on the set of p∈P(X) that satisfy the nonlinear constraint on *p* in the form $g:R_{+}^{n}\to R,\;g\left(p\right)=y$ that is

(20) $$g\left(p\right)=f\left(E_{p}\left[h\right]\right)=f(\sum_{i=1}^{n}h_{i}p_{i})=y.$$

The choice of this type of constraints is motivated by classical applications in statistical physics. For example in the Ising model in the Curie–Weiss (mean field) approximation the average energy of the spin lattice is a quadratic function of the average magnetization $E_{p}\left[s\right]$, see [14,15]. We have that

(21) $$dg\left(p\right)=f^{\prime }\left(E_{p}\left[h\right]\right)h=\left(f^{\prime }\left(E_{p}\left[h\right]\right)h_{1},\ldots ,f^{\prime }\left(E_{p}\left[h\right]\right)h_{n}\right).$$

Note that we do not take into account at this stage of the procedure the normalization constraint stipulating that we will enforce it by dividing any candidate extremum point $\hat{p}$ by $\sum_{i}{\hat{p}}_{i}$. After introducing the Lagrange function where λ is the Lagrange multiplier associated to the constraint (20)

(22) $$G\left(y,p,\lambda \right)=D\left(p,q\right)-\lambda (f\left(E_{p}\left[h\right]\right)-y)$$

we see that the candidate extrema are the solutions (p,λ) for given *y* of (here i=1,…,n)

(23) $${\left(G_{p}\right)}_{i}=\ln\frac{p_{i}}{q_{i}}+1-\lambda f^{\prime }\left(E_{p}\left[h\right]\right)h_{i}=0,\;G_{\lambda }=f\left(E_{p}\left[h\right]\right)-y=0$$

that is, setting $q_{i}=e^{k_{i}}$, we have to face a trascendental equation for the unnormalized probability

(24) $$p_{i}=c\;e^{\lambda f^{\prime }\left(E_{p}\left[h\right]\right)h_{i}+k_{i}},\;f\left(E_{p}\left[h\right]\right)=y.$$

After normalization, (24)_1_ becomes

(25) $$p_{i}=e^{\lambda f^{\prime }\left(E_{p}\left[h\right]\right)h_{i}+k_{i}-\psi \left(\lambda ,p\right)},\;e^{\psi (\lambda ,p)}=\sum e^{\lambda f^{\prime }\left(E_{p}\left[h\right]\right)h_{i}+k_{i}}.$$

Let us denote with $f^{\leftarrow }\left(y\right)\subset R$ the set of pre-images of *y* along *f* (see, e.g., Figure 1 below)

(26) $$f^{\leftarrow }\left(y\right)=\left\{\eta \in R\;:\;f\left(\eta \right)=y\right\}=\left\{\eta_{1},\ldots ,\eta_{\alpha },\ldots ,\eta_{A}\right\},\;\eta_{\alpha }=\eta_{\alpha }\left(y\right)$$

where we have supposed that, for every *y*, $f^{\leftarrow }\left(y\right)$ is a *finite* set of cardinality A(y)<+∞. The crux is that we can substitute the constraint $f(E_{p}\left[h\right])=y$ in (24)_2_ with the following equivalent one

$$f\left(E_{p}\left[h\right]\right))=y\;⟺\;E_{p}\left[h\right]\in f^{\leftarrow }\left(y\right)$$

therefore we can describe the—possibly non-unique—solution (25) of the extremum problem (23) as

(27) $$p_{i}^{\alpha }=e^{\lambda f^{\prime }\left(\eta_{\alpha }\right)h_{i}+k_{i}-\psi \left(\lambda ,\alpha \right)},\;e^{\psi (\lambda ,\alpha )}=\sum e^{\lambda f^{\prime }\left(\eta_{\alpha }\right)h_{i}+k_{i}}$$

where α=1,…,A(y), showing that the candidate solution belongs to an exponential family M(h,k). Note that in Information Geometry, the critical points of the MEP extremum problem are computed as *geodesic projections* over a submanifold which is an exponential family and multiplicity of solutions are related to the non-uniqueness of the geodesic projection, see in [1,15].

Note that *where* $f^{\prime }\left(\eta_{\alpha }\right)\neq 0$ setting $\lambda f^{\prime }\left(\eta_{\alpha }\right)=\theta_{\alpha }$ the solution (27) can be given the standard form (see in [1,14]) of MEP solution

$$\;{\hat{p}}_{i}=e^{\hat{\theta }h_{i}+k_{i}-\psi \left(\hat{\theta }\right)},\;\hat{\theta }\left(\eta \right)=\varphi_{\eta }\left(\eta \right)$$

with *linear* constraint $E\left[h\right]=\eta_{\alpha }$, hence (25) becomes

(28) $$p_{i}^{\alpha }=e^{\theta_{\alpha }h_{i}+k_{i}-\psi \left(\theta_{\alpha }\right)},\;e^{\psi (\theta_{\alpha })}=\sum e^{\theta_{\alpha }h_{i}+k_{i}}.$$

The multipliers $\theta_{\alpha }=\hat{\theta }\left(\eta_{\alpha }\left(y\right)\right)$, α=1,…,A(y) are uniquely determined (see (10)) by the equation

(29) $$\psi_{\theta }\left(\theta \right)=\eta \;i.e.\;\hat{\theta }\left(\eta \right)=\varphi_{\eta }\left(\eta \right)$$

for $\eta =\eta_{\alpha }\left(y\right)$ and accordingly we can compute the multipliers λ as

(30) $$\lambda_{\alpha }\left(y\right)=\frac{\hat{\theta }\left(\eta_{\alpha }\left(y\right)\right)}{f^{\prime }\left(\eta_{\alpha }\left(y\right)\right)}.$$

Note that the solution to our constrained extremization problem (28) has the form of a curved exponential family (see [1]) with respect to the discrete parameter α. We will see in the next Section 3.1 that the framework of Lagrangian submanifold is useful to describe the global picture of the solutions in case of multiple solutions.

### 3.1. The Global Picture via Lagrange Submanifold

If we set in the Lagrange function (22) (p,λ)=u, we see that for G(y,u) the set of points (y,u) satisfying the first order necessary condition for unconstrained extremum (23) is the *critical* set

$$E=\{\left(y,u\right):G_{u}\left(y,u\right)=0\}.$$

We can check if the Lagrange function G(y,u) defines a Morse family using the rank condition (3)

$$\mathrm{rk}\left(G_{yu}\;G_{uu}\right)=n+1\;\mathrm{for}\;\mathrm{all}\;\left(y,u\right)\in E$$

where in this case

(31) $$\left(G_{yu}\;G_{uu}\right)=\left(\begin{array}{ccc} 0 & \;G_{pp} & -dg^{T}\\ 1 & -dg & 0 \end{array}\right)$$

and $G_{pp}$ is the *n*-dimensional Hessian matrix (here $\delta_{ij}$ is Kronecker symbol)

(32) $${\left(G_{pp}\right)}_{ij}={\left(D_{pp}\right)}_{ij}-\lambda f^{\prime \prime }\left(E_{p}\left[h\right]\right)h_{i}h_{j}=\frac{\delta_{ij}}{p_{i}}-\lambda f^{\prime \prime }\left(E_{p}\left[h\right]\right)h_{i}h_{j}.$$

If G(y,u) is a Morse family, then by Maslov–Hormander theorem

(33) $$\Lambda_{G}=\left\{\left(y,G_{y}\right)\;\mathrm{where}\;\left(y,u\right)\in E\right\}$$

is a Lagrangian submanifold of $T^{*}R$. We claim that (33) provides a global description of the set of solutions (28). We have seen in Section 1 that a sufficient condition for the elimination of all extra parameters *u* is that $G_{uu}$ has maximal rank for all (y,u)∈E. A criterion for this is given by the following classical result in constrained optimization theory, here adapted to our notations, which express the second order sufficient condition for maxima or minima (see in [14,21] for the proof).

**Proposition** **2.** *If the symmetric matrix $G_{pp}$ in (32) is (positive or negative) definite on kerdg for (y,u)∈E, then the square matrix $G_{uu}$ in (31) has maximal rank.*

From (21), we have that for (y,u)∈E

$$\ker dg\left(p\right)=\{u\in R^{n}\;:\;f^{\prime }\left(\eta_{\alpha }\right)h\cdot u=0\}$$

and from (32), that

$$G_{pp}u\cdot u=\left(\sum_{i}\frac{u_{i}^{2}}{p_{i}}\right)-\lambda f^{\prime \prime }\left(\eta_{\alpha }\right){\left(h\cdot u\right)}^{2}.$$

It is straightforward to derive from the above relations that the two cases below hold

$$\left\{\begin{array}{c} f^{\prime }\left(\eta_{\alpha }\right)\neq 0\;\;\Rightarrow \;\ker dg\left(p\right)=\left\{u\;:\;h\cdot u=0\right\}\;\Rightarrow \;G_{pp}u\cdot u>0\;\;\forall \;u\neq 0,\\ f^{\prime }\left(\eta_{\alpha }\right)=0\;\;\Rightarrow \;\ker dg\left(p\right)=R^{n}\;\;\;\Rightarrow \;G_{pp}u\cdot u\in R. \end{array}\right.$$

Therefore, at points (y,u)∈E where $f^{\prime }\left(\eta_{\alpha }\right)\neq 0$ the Lagrangian submanifold $\Lambda_{G}$ in (33) is transversal. At points in E where $f^{\prime }\left(\eta_{\alpha }\right)=0$, we have $dg=f^{\prime }\left(\eta_{\alpha }\right)h=0$, see (21), thus transversality is lost as—see the form of $G_{uu}$ in (31)—for these points

$$\det G_{uu}\left(p,\lambda \right)=0,\;\;and\;\;\left(y,u\right)\in E.$$

We remark that the above introduced framework is able to give the global description of the set of solutions (28), (30) in terms of the Lagrangian submanifold locally described as

(34) $$\Lambda_{f}^{\left(y\right)}=\left\{\left(y,G_{y}\right)=\left(y,\lambda (\eta_{\alpha }\left(y\right))\right)=(y,\frac{\hat{\theta }\left(\eta_{\alpha }\left(y\right)\right)}{f^{\prime }\left(\eta_{\alpha }\left(y\right)\right)})\right\}\subset T^{*}R_{y}$$

where $\lambda (\eta_{\alpha }\left(y\right))$ is given by (30). If we consider $f:E\subset R_{\eta }\to R_{y}$, y=f(η) as a local change of coordinates on M(h,k) (since *f* is locally invertible where $f^{\prime }\left(\eta \right)\neq 0$) it is easy to prove that

**Proposition** **3.** *The submanifold $\Lambda_{f}^{\left(y\right)}\subset T^{*}R_{y}$ in (34) is the image $\Lambda_{f}^{\left(y\right)}=T^{*}f\left(\Lambda_{f}\right)$ of*

(35) $$\Lambda_{f}=\left\{\left(\eta ,{\hat{\theta }}_{\alpha }\left(\eta \right)\right)\;:\eta \in E\right\}\subset T^{*}M\left(h,k\right)$$

*where ${\hat{\theta }}_{\alpha }\left(\eta \right)$ is the multiplier in (29) associated to the constraint $E_{p}\left[h\right]=\eta_{\alpha }$ and $\eta_{\alpha }\in I\left(\eta \right)=f^{\leftarrow }\left(f\left(\eta \right)\right)$.*

**Proof.**  If y=f(η) is the local change of coordinates in M(h,k), then the tangent map $Tf:TR_{\eta }\to TR_{y}$ has the local form $\left(y,\dot{y}\right)=Tf\left(\eta ,\dot{\eta }\right)=\left(f\left(\eta \right),f^{\prime }\left(\eta \right)\dot{\eta }\right)$ and the cotangent map $T^{*}f:T^{*}R_{\eta }\to T^{*}R_{y}$ has the local form

$$\left(y,\lambda \right)=T^{*}f\left(\eta ,\beta \right)=\left(f\left(\eta \right),\frac{\beta }{f^{\prime }\left(\eta \right)}\right)$$

if we want that the Liouville one-form (see above (1)) has the same canonical form $\theta_{c}\;=\;\lambda dy=\;\beta d\eta$ in the two coordinate charts. See, e.g., in [19] for a proof of this last classical result of differential geometry. □

We want to study the Lagrangian submanifold $\Lambda_{f}$ defined in (35) and its image $L_{f}=F_{D}^{-1}\left(\Lambda_{f}\right)\subset M{\left(h,k\right)}^{2}$, where $F_{D}^{-1}$ is defined in (14), whose local expression is

(36) $$L_{f}=\left\{\left(\eta ,\hat{\theta }\left(\eta \right)-\hat{\theta }\left(\eta_{\alpha }\right)\right)\;:\;\eta \in E\right\}.$$

**Figure 2:**
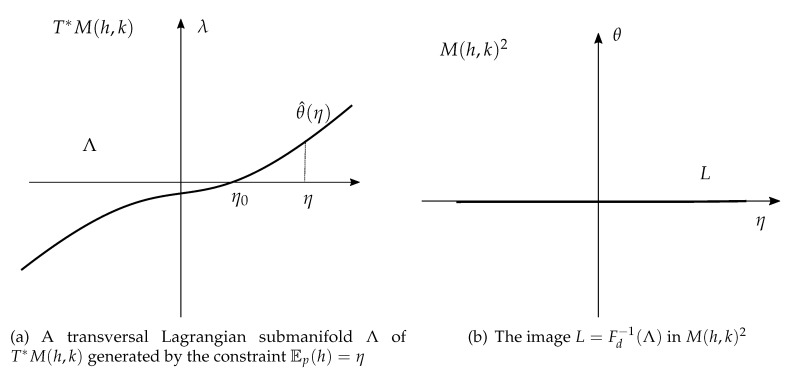
The case of a transversal Lagrangian submanifold.

First we consider the case that *f* is a globally invertible function. In this case, $I\left(\eta \right)=f^{\leftarrow }\left(f\left(\eta \right)\right)=\left\{\eta \right\}$ and $\hat{\theta }\left(\eta \right)=\varphi_{\eta }\left(\eta \right)$. The Lagrangian submanifold $\Lambda_{f}$ in (35) is the graph of the differential $\varphi_{\eta }\left(\eta \right)$ and it is transversal, see Figure 2a. Moreover, see below (9), if $\eta =\eta_{0}=E_{q}\left[h\right]$ then $\hat{\theta }\left(\eta_{0}\right)=0$. As $\psi_{\theta }\left(\theta \right)=\eta$ is invertible with inverse $\theta =\hat{\theta }\left(\eta \right)$, we have

$$\frac{d\hat{\theta }}{d\eta }\left(\eta \right)={\left(\frac{d^{2}\psi }{d\theta^{2}}\right)}^{-1}={\mathrm{var}}_{\hat{p}}\left(h\right)=E_{\hat{p}}\left[h^{2}\right]-\eta^{2}>0$$

and $\hat{\theta }\left(\eta \right)$ is a monotonically increasing function, see Figure 2a. Its image (36) is $L_{f}=M\left(h,k\right)\times \left\{0\right\}$, see Figure 2b.

**Figure 3:**
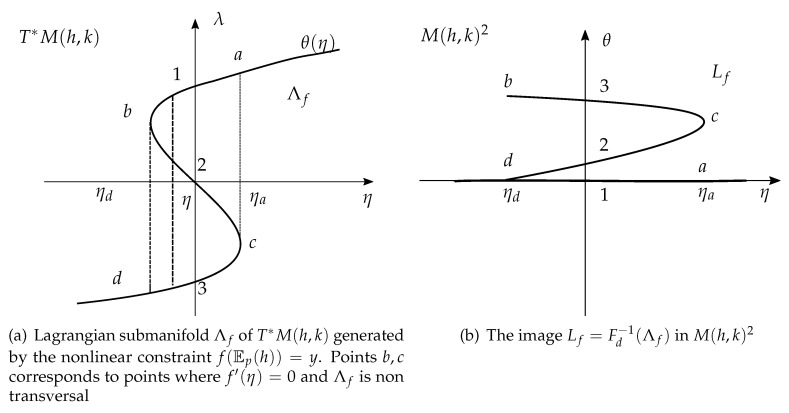
The case of a folded, i.e., non transversal Lagrangian submanifold.

If we consider a globally non invertible function *f* as the one depicted in Figure 1, then I(η) contains multiple points and $\Lambda_{f}$ is non transversal at points where $f^{\prime }\left(\eta \right)=0$, see Figure 3a. The corresponding image $L_{f}$ has multiple branches and it is not a manifold at points (b,c) where transversality fails, see Figure 3b).

### 3.2. Probability Distributions in $L_{f}$

In this section, we study the structure of the probability distributions in $L_{f}$. In the local coordinate systems (η,θ) of $M{\left(h,k\right)}^{2}$, η and $\hat{\theta }\left(\eta \right)$ describe the same probability distribution that we write for brevity as $p_{i}\left(\eta \right)=p_{i}\left(\hat{\theta }\right)$. Therefore, the probability distributions in $L_{f}$ in (36) associated to η and $\hat{\theta }\left(\eta \right)-\hat{\theta }\left(\eta_{\alpha }\right)$ are, respectively,

(37) $$p_{i}\left(\eta \right)=e^{\hat{\theta }h_{i}-k_{i}-\psi \left(\hat{\theta }\right)}$$

and, see (18),

(38) $$p_{i}\left(\hat{\theta }-\hat{\theta }\left(\eta_{\alpha }\right)\right)=p_{i}\left(\eta \right)\frac{e^{-\hat{\theta }\left(\eta_{\alpha }\right)h_{i}}}{\sum_{i}p_{i}\left(\eta \right)e^{-\hat{\theta }\left(\eta_{\alpha }\right)h_{i}}}.$$

Setting

$${\tilde{p}}_{i}\left(\eta_{\alpha }\right)=\frac{e^{-\hat{\theta }\left(\eta_{\alpha }\right)h_{i}}}{Z(\lambda )},\;Z\left(\lambda \right)=\sum_{i}e^{-\hat{\theta }\left(\eta_{\alpha }\right)h_{i}},$$

the above (38) can be rewritten as the discrete version of (19), that is,

(39) $$p_{i}\left(\hat{\theta }-\hat{\theta }\left(\eta_{\alpha }\right)\right)=\frac{p_{i}\left(\eta \right){\tilde{p}}_{i}\left(\eta_{\alpha }\right)}{\sum_{i}p_{i}\left(\eta \right){\tilde{p}}_{i}\left(\eta_{\alpha }\right)}.$$

This last formula can be interpreted as follows; let *A* and *B* be two independent random variables A,B: Ω→X, where X={1,…,n} is the discrete state space, described by the probability distributions $p_{i}$ and ${\tilde{p}}_{i}$, respectively (for example, *A* and *B* describe two dices with *n* faces). Then, $\sum_{i}p_{i}{\tilde{p}}_{i}$ is the probability that *A* and *B* are found in the *same* state and

$$Prob\left(A=i,B=i|A=B\right)=\frac{p_{i}{\tilde{p}}_{i}}{\sum_{i}p_{i}{\tilde{p}}_{i}}$$

in (39) is the conditional probability that *A* and *B* are found in the state *i* provided that they are found in the same state. Note that for $p_{i}\left(\eta \right)$ in (37) we have $e^{k_{i}}=q_{i}$, thus (37) can be rewritten as

$$p_{i}\left(\eta \right)=q_{i}\frac{e^{\hat{\theta }h_{i}}}{\sum_{i}q_{i}e^{\hat{\theta }h_{i}}}=\frac{q_{i}\tilde{p}\left(\hat{\theta }\right)}{\sum_{i}q_{i}{\tilde{p}}_{i}\left(\hat{\theta }\right)}$$

and (39) above is equal to

$$p_{i}\left(\hat{\theta }-\hat{\theta }\left(\eta_{\alpha }\right)\right)=\frac{q_{i}p_{i}{\tilde{p}}_{i}}{\sum_{i}q_{i}p_{i}{\tilde{p}}_{i}}=Prob\left(A=i,B=i,C=i|A=B=C\right)$$

where A,B,C are described by $q_{i}\;$, $p_{i}={\tilde{p}}_{i}\left(\hat{\theta }\left(\eta \right)\right)\;$, ${\tilde{p}}_{i}={\tilde{p}}_{i}\left(\hat{\theta }\left(\eta_{\alpha }\right)\right)$.

## 4. Discussion

Canonical coordinates η and θ associated to an exponential family M(h,k) are dually flat coordinates with respect to the duality defined by the canonical divergence. With respect to these coordinates, a generalization of the Pitagorean theorem is proved in Information Geometry which provides a generalized formulation of the Maximum Entropy Principle with linear constraints as a geodesic projection problem (see [2]). Multiplicity of the solutions $\hat{\theta }\left(\eta \right)$ of the Maximum Entropy problem are due to the non uniqueness of the projection. In this paper, we have shown that the set of couples $\left(\eta ,\hat{\theta }\left(\eta \right)\right)$ defines a transversal Lagrangian submanifold Λ of $T^{*}M\left(h,k\right)$, and we have seen with an example that if nonlinear constraints are considered the set of possible multiple solutions to the Maximum Entropy problem is globally described by a folded (i.e., a possibly non-trasversal) Lagrangian submanifold $\Lambda_{f}$. We have computed their pull-back to the square manifold $M{\left(h,k\right)}^{2}$ via the map $F_{D}^{-1}$. We think that this framework offers a complementary view to the generalized Pitagorean Theorem. We plan to address in a subsequent paper a generalization of the theory presented here to a more general form of nonlinear constraint.
